# Editorial: The Wildlife Gut Microbiome and Its Implication for Conservation Biology

**DOI:** 10.3389/fmicb.2021.697499

**Published:** 2021-06-21

**Authors:** Lifeng Zhu, Jianjun Wang, Simon Bahrndorff

**Affiliations:** ^1^Colleges of Life Science, Nanjing Normal University, Nanjing, China; ^2^State Key Laboratory of Lake Science and Environment, Nanjing Institute of Geography and Limnology, Chinese Academy of Sciences, Nanjing, China; ^3^Department of Chemistry and Bioscience, Aalborg University, Aalborg, Denmark

**Keywords:** microbiome, adaptation, host-microbiome, reintroduction, conservation

It has long been recognized that microbial symbionts can affect hosts in many ways [e.g., (Turnbaugh et al., 2006; Frank et al., 2007; Wen et al., 2008; Engel and Moran, 2013)], and in recent years much emphasis has thus been put into understanding the importance of microbiomes of animals. This has happened together with the advancement in high-throughput sequencing, which has made it feasible and economically viable to identify microbiomes among and within hosts [e.g., (Caporaso et al., 2012)]. These factors together, paved the way for a new area of research in conservation biology focusing on the importance of host-microbiome associations for endangered species and importance for conservation efforts. Early studies highlighted the importance of the host-microbiome for conservation efforts (Amato et al., 2013; Jani and Briggs, 2014) and in the years after a number of studies have addressed the conceptual aspects of the host-microbiome and conservation efforts (Bahrndorff et al., 2016; Stumpf et al., 2016), which was further elaborated on in more recent papers (Jiménez and Sommer, 2017; Hauffe and Barelli, 2019; Trevelline et al., 2019; West et al., 2019). Since then, the number of studies that have addressed different aspects of host-microbiome associations and conservation efforts has almost been exponentially increasing (“Conservation” and “Microbiome” as keywords in Web of Science). This has led to a tremendous amount of data on host-microbiomes across multiple host phyla [e.g., (Zhu et al., 2011; Bourne et al., 2013; Jani and Briggs, 2014; Cheng et al., 2015; Galac et al., 2016; Chen et al., 2017; Bahrndorff et al., 2020)]. Many of these studies have typically looked at host-microbiomes of endangered species and comparing groups that are affected or not by anthropogenic disturbances (e.g., comparing animals from the wild with individuals held in captivity, groups exposed to habitat fragmentation, or dietary shifts) (Menke et al., 2017; Jia et al., 2018; Han et al., 2019; van Leeuwen et al., 2020). From these studies, it is clear that anthropogenic disturbances will in most cases affect the host-microbiome, but it is often unclear how and if hosts are affected by such changes.

The aim of the Frontiers in Microbiology Research Topic “The Wildlife Gut Microbiome and Its Implication for Conservation Biology” was to collect state-of-the-art articles on wildlife gut microbiome composition and function, and importance for conservation biology. We hereby provide an overview of this Frontiers in Microbiology topic, which includes 18 original articles and 3 review articles.

## Reintroduction and Importance of Host-Microbiomes

One of the effective strategies to conserve endangered species with small population sizes is through reintroduction of individuals kept in captivity into the wild. This can introduce some challenges as the captive animals are often kept under conditions that can be very different compared with their natural environments (e.g., different diets). The animal gut microbiome plays an important role in nutrition intaking and host health, and can increase the ability to adjust from conditions under captivity to natural conditions. Several papers address these important issues in this special issue (e.g., Sun et al.; Tang G-S. et al.; Tang L. et al.; Yang et al.; Prabhu et al.; Ning et al.). In most studies there is a clear effect of keeping animals in captivity or from domestication. However, what is particularly interesting is the fact that responses are species specific, and that some species show a less diverse microbiome when held in captivity and the opposite for other species (e.g., Tang G-S. et al.; Tang L. et al.; Sun et al.). For example, Tang et al. find that individuals of the endangered crocodile lizards (*Shinisaurus crocodilurus*) held in captivity had an increased community richness compared to wild populations.

The fact that being held in captivity affect host-microbiome associations and also the functional role of the microbiomes suggests that release programs should address the microbiomes before individuals are being located into the wild again. With focus on host-microbiomes of individuals being released into the wild it is possible to increase the likelihood of successful release. This is exemplified by Yang et al. showing an effect of prerelease diet training on the microbiome and fitness of the endangered Yangtze sturgeon (*Acipenser dabryanus*) after releasing into the wild environment. The key point in this study is the natural diet training for these translocated individuals, that may benefit for their local adaptation.

## Host-Microbiomes and Their Functional Role

The close association of hosts and their microbiomes has naturally resulted in a number of studies addressing the functional role of the microbiome [e.g., (Bahrndorff et al., 2018; Antwis et al., 2019; Guo et al., 2019; Wang et al., 2019)], adding much-needed information to the ecological aspects of conservation biology. In the present topic many of the studies address the functional role of the host-microbiomes (e.g., Ning et al.; Tang G-S. et al.; Tang L. et al.; Sun et al.). This is particularly interesting from a conservation point of view as this may help explain for instance the poor reintroduction attempts of endangered species. For example, Sun et al. shows that the bacteria of wild individuals of the alpine musk deer (*Moschus chrysogaster*) are enriched for genes involved in e.g., enzyme digestion of cellulose. Similarly, Ning et al. compare the gut microbiome composition and function between individuals held in captivity and wild populations of a large carnivore (Amur tiger *Panthera tigris altaica*). They find a significant difference in the gut microbiome and metagenome, and analysis further reveals 12 KEGG pathways (e.g., immune system and cell mobility) that are enriched in the wild population gut microbiome, but only two pathways are enriched in the captive population gut microbiome. The difference in the diet and living environment may explain the difference in the gut microbiome of Amur tiger in this study.

## Environmental Variables and The Evolution of Host-Microbiome Associations

Environmental variables are important in determining species composition and distributions, but may also affect host-microbiome associations (e.g., Prabhu et al.; Schellenberg and Clarke; Kivistik et al.; Sepulveda and Moeller; Zhu et al.; Zhu et al.). Host-microbiome associations may also prove particularly relevant for organism's ability to adapt to changes in environmental conditions as the microbiome can affect host phenotypes and since bacteria will respond to environmental changes on a much shorter timescale compared to the host. For example, Sepulveda and Moeller discuss how temperature affects host-microbiomes and how extreme events may affect the host phenotype, and Zhu et al. find that duration and thermal acclimation temperature significantly affect the microbiome of the Chinese giant salamander (*Andrias davidianus*). Kivistik et al. test the importance of salinity on the bacterial community of *Theodocus dluviatilis* and the ability of populations to respond to changes in salinity. Together, these results suggest that environmental variables can shape host-microbiomes and affect host fitness. The long-term evolutionary aspects of host-microbiomes is addressed by Becker et al. looking at the gut microbiome community in two Old World vulture species [the Griffon (*Gyps fulvus*) and the Egyptian vulture (*Neophron percnopterus*)]. The gut microbial diversity is found to be significantly different between these two vulture species, which may reflect evolved feeding ecologies. The novelty in this study is to reveal the necessity to adjust the captive husbandry based on their wild food composition, although they have a closed phylogenetic relationship. This is important for the recovery plans of endangered vulture species due to the health of reintroduced individuals, but also that recovery plans should include species-specific strategies. The co-evolution between marsupials and their gut microbiomes is also addressed in a review by Chong et al. They highlight the dominance of Firmicutes, Bacteroidetes, and Proteobacteria in the marsupial gut microbiome and that different marsupials display a species-specific gut microbiome, which further supports the effects on the gut microbiome by host phylogeny.

## Future of Wildlife Gut Microbiome in Conservation Biology

The importance of host-microbiome associations for conservation biology and recent advances in microbiome studies is shown in the published studies of this topic. However, the advances in host-microbiome studies also raises new issues and questions, such as the importance of sequencing depth, bioinformatic analyses, and quality of databases, which for example can affect the ability to identify and predict the functional role of bacteria. While many studies are, and will also be in the future, use marker-based sequencing approached (for many good reasons), whole-genome approaches may be increasingly used in future studies (e.g., Ning et al.; Sun et al.; Tang L. et al.; Mittal et al.; Roth et al.). Further, a holo-omic framework (Limborg et al., 2018; Zepeda Mendoza et al., 2018) could prove especially useful to further elucidate host-microbiome interactions and the importance of microbiomes for conservation efforts (Roth et al.; Ning et al.). The use of e.g., metagenomics will also lead to further focus on analysis of large datasets and development of new bioinformatic tools (Dong et al.). At the same time, it is clear that we need to go “back to basics,” using traditional microbiological approaches to obtain further knowledge on the functioning of specific strains under more controlled conditions and to promote the applied use of bacteria, e.g., use of probiotics in conservation biology. Further, validation and development of non-invasive sampling techniques for microbiome studies will strengthening the use of microbiome studies in conservation biology. It is also likely that microbiomes will play a larger role in future conservation biology, where the conservation of endangered species will be more dependent on populations held in captivity. Since microbiomes can affect host fitness, an obvious question is also whether such effects may result in changes at the population level affecting demography of endangered species. Altogether, this asks for further large-scale collaborative studies and efforts across research groups and other governmental organizations and NGOs to address and use microbiome research in the conservation of endangered species.

## Author Contributions

All authors listed have made a substantial, direct and intellectual contribution to the work, and approved it for publication.

## Conflict of Interest

The authors declare that the research was conducted in the absence of any commercial or financial relationships that could be construed as a potential conflict of interest.
